# Understanding Patient Needs Regarding Adverse Drug Reaction Reporting Smartphone Applications: A Qualitative Insight from Saudi Arabia

**DOI:** 10.3390/ijerph18083862

**Published:** 2021-04-07

**Authors:** Lamyaa M. Kassem, Bushra Alhabib, Khaledah Alzunaydi, Maryam Farooqui

**Affiliations:** Department of Pharmacy Practice, Unaizah College of Pharmacy, Qassim University, Unaizah 51911, Saudi Arabia; bushra.e.alhabib@gmail.com (B.A.); khaledah_abdullah@hotmail.com (K.A.); m.farooqui@qu.edu.sa (M.F.)

**Keywords:** adverse drug reactions, reporting system, technology, patient, healthcare professionals, KSA, qualitative

## Abstract

Background: A pragmatic shift in the healthcare sector characterized by moving from curative to preventive approaches highlights the role of pharmacovigilance in patient safety. There have been few published studies on patient reporting of adverse drug reactions (ADRs) in Saudi Arabia. This qualitative study aims to explore the community opinions and the need for patient-friendly smartphone applications (SPAs) to enhance their participation in ADR reporting. Methods: Purposeful sampling was followed to recruit study participants, a semi-structured interview guide was used to conduct interviews, and the saturation was reached after the 13th interviewer; no new information was obtained after two subsequent interviews. All the interviews were audio-recorded, transcribed verbatim, and analyzed by means of a standard content analysis framework. Results: As per the WHO guidelines, eleven participants were aware of the term “ADR”. All the participants denied receiving any prior education and attending events about ADRs and were unaware of the Saudi FDA-ADR reporting systems. The use of technologies such as SPAs has been widely accepted with a high level of concern for data confidentiality and privacy. Conclusions: These findings point out the need to build patient-oriented educational programs to increase their awareness of ADR reporting and to prioritize the use of artificial intelligence (AI) to be integrated in the Saudi healthcare system to develop future SPAs for improving both patient safety and signal detection of ADRs.

## 1. Introduction

The World Health Organization (WHO) and the European Union recognized the importance of patient self-reporting of ADRs [1]. Historically, spontaneous reporting (SR) of ADRs was performed only by healthcare professionals; however, patient engagement is the latest step towards more effective pharmacovigilance (PhV). Patient reporting of ADRs has been described in the literature as a noteworthy resource of collecting new data about the safe use of drugs. The patients’ first-hand reports have been issued in Europe since 2010 according to the European PhV legislation (Directive 2010/84/EU) which introduced a new framework for drug surveillance and proposed valuable changes to improve drug safety [2]. The patient ADR reporting system is well-established in some developed countries but has not yet been adopted in most countries including the developing countries [1]. Policymakers around the world are making tremendous efforts to develop an innovative healthcare technology holding a considerable promise for the future of PhV [3,4]. Over the last decades, several patient-friendly SPAs have been developed and incorporated into the patient day-to-day life in different healthcare and services sectors. The Food and Drug Administration (FDA) has expressed increased concerns over health informatics including SPAs, and the entities concerned decided that all health applications have to be monitored and regulated by the FDA. Christy Foreman, FDA Director of the Product Assessment Office, said, “The FDA finds it necessary to take a balanced approach to mobile medical devices, which encourages innovation and ensures proper patient safety.” Examples of the best FDA-approved and -certified patient-friendly mobile health SPAs are as follows: AliveCor, Well Doc’s Diabetes Boss, Welch Allyn iExaminer Adapter Ophthalmoscope, and AirStrip ONE [5]. Patient reporting adds new information on ADRs that are not widely available otherwise. This can help improve the legislative decision-making processes. A systemic review of patients reporting to the PhV system conducted by Inácio et al. (of a total of thirty-four trials) showed that patient reporting is useful in providing a detailed description of ADRs where patients often explain the extent and impact of ADRs on their daily lives [1]. As a part of the WEB-RADR pharmacovigilance project, WEB-RADR introduced mobile applications to enable patients, caregivers, and healthcare professionals to report adverse drug reactions (ADRs) and receive medication updates and news notifications. The Med Safety smartphone application released by the collaboration between WEB-RADR and the WHO, the Medicines and Healthcare products Regulatory Agency (MHRA), and the Uppsala Monitoring Center (UMC) has been launched in Burkina Faso, Zambia, Armenia, Ghana, Ethiopia, Botswana, Ivory Coast, and Uganda. There are also some country-specific SPAs such as MHRA (UK), Lareb (the Netherlands), and Halmed (Croatia), and they constantly contribute a lot to the flourishing mobile health industry [6]. In general, smartphone applications aim to benefit patients and thus improve treatment outcomes. Several technologies have been successfully used to achieve those therapeutic goals in terms of communication with patients [7] and monitoring and improving patient compliance [8] like asthmatic patients’ adherence [9] and monitoring [10]. Smartphone applications are also used for patient education [11] and diabetic patient care [12].

Convergence of healthcare and information technologies provides promising new trends to improve access to high-quality healthcare services at affordable prices, thus helping meet evolving customer needs [13]. To achieve this goal, Saudi Arabia is creating a new, independent, and evidence-based health technology assessment (HTS) agency to help it optimize health benefits by using the resources efficiently. The KSA is undergoing a major healthcare transition to achieve its own new national “Vision 2030” [14]. The Ministry of Health (KSA) utilized a number of public-targeted applications to enforce the COVID-19 curfew and track contagious cases and increase public safety, such as Tawakkalna, Tabaud, Mawid, Sehaty, and Tetamman. Those applications have all been successfully used by all the citizens in Saudi Arabia [15,16]. A patient ADR reporting form (online) was developed by the Saudi FDA several years ago but has not been promoted yet [17,18]. Given the monthly reporting list of Saudi Food and Drug Authority (SFDA) hospital ADRs (May 2020), only 42 out of all the Saudi hospitals reported 1819 ADRs, with six hospitals reporting hundreds of ADRs, nine hospitals reporting up to 100 ADRs, and only one ADR reported by most hospitals in one month. SFDA ADR lists released by the agency’s official LinkedIn page do not contain a single patient self-report [19]. The latest published statistics (2019) indicated that the number of smartphone users in Saudi Arabia is estimated to reach 28.8 million, which the higher authorities have taken into account when introducing and generalizing new laws to meet the new population needs and the 21st century technologies; moreover, the number of smartphone users is expected to increase further in the Middle East [20]. Despite the increasing number of smartphone users in the KSA and the Middle East and the presence of well-established pharmacovigilance centers in the KSA as well as in many Gulf countries, the ADR reporting is dependent on HCPs and hospitals and patient self-reporting has not been highlighted yet. Patients’ involvement in reporting improves signal detection and both drug and patient safety. The patient needs regarding portable ADR reporting tools have not been well-explored among the Saudi population and there are no previously published studies to address their needs and highlight the importance of developing a patient-friendly ADR reporting smartphone application. With regard to launching a new patient care service that improves medication safety, we conducted the current study. The objective of the study was to address the actual patients’ and community needs in the context of patient-friendly ADR reporting. Through this qualitative study, we can deeply explore the real needs of the patients and population regarding ADR reporting and their awareness, intentions, and attitudes towards using information technologies (IT) and technological smartphone applications.

## 2. Methods

### 2.1. Study Setting and Design

This qualitative study was conducted at Unaizah College of Pharmacy (UCP), Qassim Region, KSA. Adverse drug reactions and attitudes towards using technology are likely to be perceived contrarily by different patients; the social constructivism paradigm was adopted [21]. The typical examples of elements found important by participants were illustrated by quotes.

### 2.2. Ethical Considerations

This study was approved by the Qassim University’s Research Ethics Committee (No. 20-05-06). Prior to the interview, information regarding their voluntary participation, anonymity, and right to withdraw at any time was provided to all the participants. The authorized informed consent to the recording and use of transcripts of the interviews for research purposes was received from each participant. The data were analyzed after participants’ approval of their respective interview transcripts. All the data were properly transcribed, safely stored, and read by authors only to maintain privacy and confidentiality.

### 2.3. Sampling and Recruitment

For the present study, participants were selected from Unaizah College of Pharmacy (UCP) between January and March 2020. Staff members aged between 18 and 65 years including both administrative and academic staff were invited to participate. Participants who were familiar with smartphone applications and had previous personal, family, or friends’ experience with ADRs were specifically included. The purposeful sampling technique was adopted to look for information-rich cases. We approached the participants at their offices and briefed them about the study objectives. The participants were also informed about the nature of qualitative interviews and the consent was obtained to record the interviews. Those who declined to be recorded were excluded. Since the staff members were from different age groups with different socioeconomic status and educational background, we believe that we were able to recruit a representative population. All the participants were provided with a study information sheet in Arabic (the national language), while verbal information was also provided as per participant’s request. Sampling continued until the theoretical saturation was believed to have been achieved and no new information was added. Two subsequent interviews were conducted to confirm saturation; however, they were not included in the final analysis. The interview time and place were chosen according to the participant’s preferences.

### 2.4. Study Tool

For the data collection purpose, a comprehensive literature review [22,23] and consultations with experts in the field were undertaken to develop a semi-structured, open-ended questions interview guide (Table 1). The interview guide consisted of possible questions to assess patient expectations, understanding, and experience with ADRs. Most of the questions were open-ended as they offer the best opportunity to represent views of the participants and allow a deeper grasp of the related issues. After several rounds of consultations, the preliminary draft of the interview guide was reviewed by the authors and modified accordingly. To ensure that the set of questions developed was useful for objective information retrieval, pilot interviews were conducted with five participants. Those included during piloting were excluded from the final analysis. The draft interview guide was updated again based on the specific observations during the pilot interviews. Cohen’s kappa coefficient (κ) was used to ensure the degree of agreement [24]. Participants’ demographics and other relevant data such as the type of ADRs were also recorded in a separate datasheet.

### 2.5. Procedure and Interview Process

Face-to-face open-ended interviews were conducted in Arabic by the principal investigator (L.K.). The other two researchers (B.H. and K.Z.) attended the interviews to take notes and to facilitate the interview process. Interviews were audio-recorded. Verbatim transcriptions were in formal Arabic and translated to English for individual interview sessions following forward and backward revision between two authors (L.K. and M.F.) with the help of an English expert in English translation. Transcripts were shared with the respective study participants for their approval.

### 2.6. Data Coding and Analysis

Finally, after the receipt of verbatim transcripts from the participants, a thematic content analysis framework was used to analyze the data and assist in identifying evolving categories (themes). All the themes initially identified by the principal investigator were examined by an independent experienced qualitative researcher. The parts of transcripts related to the study were chosen as meaning units. As per Graneheim and Lundman, meaning units are described by their content and context as “words, sentences, or paragraphs containing aspects of the other” [25]. The units of meaning were stratified and counted, and codes were then categorized according to the particular meaning of the text (Table 2) [25]. Coding and categorization were frequently discussed within the study team to verify validity and adequacy of codes and categories.

## 3. Results

### 3.1. Demographic Data of the Participants

A total of 15 participants (P1–15) aged between 18 and 65 years (mean = 34 years), including 9 (60%) females and 6 (40%) males, were interviewed. Table 3 demonstrates the detailed demographic distribution of the participants.

### 3.2. Main Themes

Thematic content analysis revealed three major themes: (1) experience with ADRs, (2) identification and needs of participants regarding ADRs, and (3) awareness of ADR reporting systems and opinions on the use of technology in ADR reporting. Seven subthemes were as follows: (i) familiarity with ADRs, (ii) ADR experiences and actions taken, (iii) certainty of the symptoms, (iv) prior knowledge about ADRs, (v) awareness of international and the Saudi FDA adverse drug reaction reporting (SFDA-ADR) systems, (vi) opinions on the role of information technology (IT) in ADR reporting, and (vii) preference to have a free or paid ADR supporting service.

#### 3.2.1. Participants’ Experiences, Identification, and Needs

When the patients were asked if they had suffered ADRs, not all the participants had ADRs on their own, but they were all ready to talk about them because they observed ADRs as a usual problem among their family members and friends receiving medication.

P4: “Not me, I heard from my mother that after taking her antihypertensive medication, she had a cough that was never relieved even with an inhaler.”

P10: “No, personally, it has never happened to me, but I’ve seen this situation many times with my family and friends who would go to the prescriber or just quit taking the medication because of the seriousness of the side effect.”

#### 3.2.2. Meaning of ADRs

They all describe ADRs with the same interpretation as the WHO definition of ADRs.

P4: “Side effects are harmful effects of drugs, which sometimes I can’t differentiate from the symptoms that may be caused by my illness.”

P7: “Nasty unpleasant drug effects that make the patient dissatisfied with treatment in such a way that they can quit all medication.”

P14: “Any drug has benefits and risks and, if consumed in a larger amount than required, will cause bad effects.”

#### 3.2.3. ADR Experiences

Participants provided varying levels of experience with ADR severity, ranging from mild to serious. Some experienced minor ADRs which they managed by changing to other therapies, but others (three participants) quitted taking drugs and started looking for herbal medicines.

P12: “I assume that if I stop taking the recently used drug, this unwanted effect will resolve.”

P13: “I believe that ADRs are not as severe as the patient’s condition and if they are certain to harm the patient and worsen health, the doctor surely will not prescribe such a drug to the patient.”

#### 3.2.4. Severity of ADRs

Some patients described the fact that ADRs affect compliance, as carrying medicine along or swallowing it was difficult due to the fear of experiencing ADRs. Finally, some mentioned that despite recognizing that these ADRs are not likely to be risky, they nevertheless have faith in the physician intervention. The decision to proceed or quit using the medicine after a patient encountered ADRs varied depending on the severity of the ADR. The patients used to receive their medication details from the insert or the Internet or by seeking help of doctors or pharmacists at hospitals and pharmacies.

P3: “Yeah, after using some analgesics, I used to have abdominal pain, and I read that it’s a normal analgesic effect if I take them on an empty stomach, so I started taking them only after a meal.”

P5: “Ammm, don’t remind me of this incident, my mother was about to die after a penicillin injection, it was a very difficult time until she was saved at the hospital, I ‘m scared if me or anyone in my family gets sick just because of the fact that we’re going to take medications.”

P10: “Sure, muscle pain and extremely dry mouth after about six months of using cholesterol medication Crestor and I went to a doctor who forwarded me to another doctor.”

#### 3.2.5. ADR Counseling

When we asked the participants if they had already received or engaged in patient education services, activities, or awareness campaigns about ADRs, almost all (12) the participants claimed that they had not received or attended any events regarding patient education on ADRs; they indicated that their doctors only discussed the ADRs after they had already occurred. The patients thought that perhaps implementing some patient education programs about ADRs may result in better patient compliance and bring a positive aspect to medication safety. The participants’ responses thus reflect their need for counselors or healthcare professionals (HCPs) to assist them with ADRs.

P6: “No, only after I returned back to my physician, he told me that these drowsiness and continuous nausea are usual side effects with contraceptive pills which I wasn’t comfortable with and I decided to use an IUD, and then I asked him if there were any adverse effects if I used an IUD, he said no, but after using an IUD, my cycle became so heavy with abdominal cramps, my sister said it was normal after an IUD, and I’m not sure it might be natural IUD action to prevent pregnancy or it was a usual side effect.”

P9: “No, there’s always no one concerned in educating me about the adverse effects of our prescribed medications, they can only teach us how to use them.”

P10: “I attended one adverse drug event at a mall.”

#### 3.2.6. Participants’ Awareness of the SFDA Adverse Drug Reaction Reporting System

Most of the participants (9) were unaware of such health service and some were genuinely surprised to know the existence of online SFDA-ADRs patient reporting system which demonstrates a lack of promotion for the ADR reporting system for patients.

P3, P4, P5, P10, P13: “I have no idea.”

P4: “Mmm.... Is there a way I can disclose my adverse reactions?”

P6: “Are you sure we have this service in the KSA, if it’s there, why doesn’t anyone address the patients so they can benefit?”

P14: “Yes, I think Europe has a Yellow Card reporting system.”

P15: “Yeah, I still call 937, I think it is the only service that the Saudi Ministry of Health can help with, save time and provide me with a reliable service.”

#### 3.2.7. Opinions on the Use of Technology in ADR Reporting

The majority of the participants (11) liked the concept of using technology such as smartphone applications to get treatment for their ADRs; only two participants declined to use technology and expressed an interest in seeking face-to-face assistance by going to a healthcare setting; these two participants were over 60 years old, which explains their resistance to coping with technology.

P1: “Good and useful, it will help reduce the risk and incidence of adverse effects on patients as it will provide advice to patients with medications.”

P6: “Woow, will help reduce patient anxiety, they will be able to seek help from doctors, pharmacists anytime.”

P7: “I think it will help people who suffer and worry about the harmful effect of their medicines.”

P10: “It will help patients and reduce congestion in hospitals and attempts to take appointments to see doctors again.”

P12: “Well, technology has become an essential part of our day, and a rapid answer to our health problems via phone is good.”

P13: “I think it’s going to be very good because it will promote the century of speed and help a lot of people.”

P15: “I don’t like technology, and I don’t feel comfortable with it, and I prefer to have a phone call service that makes me feel safer.”

#### 3.2.8. Preference to Have a Free or Paid ADR Supporting Service

As expected, only few participants (3, 20%) accepted the idea of having a paid ADR reporting service on their cell phones but preferred it as a free service; more than half (8, 53%) of the participants preferred to have a free service, while two (13%) participants agreed to pay for the service if they would receive postal prescription care which also reflects their needs to receive educational advice from healthcare professionals regarding proper and safe use of medication.

P1: “Of course, if free, but if costs a little, no problem.”

Another participant despite being willing to pay for such services preferred to have it free.

P11: “I can pay for it, but I would like a free service.”

It is also noticeable that patient’s monthly income status could be a confounding factor in accepting or rejecting such services.

P12: “No, I won’t use it if it is paid because I can contact the doctor or a pharmacist, they will direct me for free. Free service will be more suitable for low-income patients.”

P10: “Depending on the need, for example, some people cannot pay, so the service should be free, and there are people who can pay fees. I suggest that fees are optional, maybe to receive other services through the app.”

If paid service, patients prefer to receive some extra services in addition to reporting of ADR. Although no further information could be obtained in the regard it could be assumed that patient may expect further advice on what to do next after reporting ADR.

P13: “If just reporting, a small fee can be charged, or it should be a free service, and in case of providing me with extra services other than reporting, I think I should pay a fee.”

P13: “If the patient reports ADRs, better if they are not charged, but if the patient can receive additional services such as follow-up or monitoring, I think it should be paid.”

Since the Saudi Ministry of Health offers a hotline service for patients to contact in case of emergencies, some of the participants preferred to upgrade the same service with ADR reporting facilities.

P15: “I prefer it free, because we have a hotline number (937) which is a free service provided by the Ministry of Health that we can ask about any patient problems.”

Interestingly, a perception that a paid service would be more efficient and effective than the unpaid one was observed.

P4: “I believe the paid service is going to be more professional.”

P9: “I wish the service to be charged so it can remain in place for long, and I believe that a small fee can be applied so that everyone can afford it.”

## 4. Discussion

With the growing dependence on medication therapy as the primary intervention for most illnesses, patients are exposed to a potential harm of their medications. Successful therapy is to achieve the required medication treatment outcomes with the least possible ADRs or toxicity which emerge in the field of medication safety and related aspects. Technology plays a great role in the way Saudi Arabia successfully manages COVID-19 where most governmental services have been smoothly transformed to remote work using technology and smartphone applications which support our idea of the need in developing a patient-friendly tool for ADR reporting. The widespread use of mobile phones among all genders and ages has paved the way for the future adoption of this technology. The future objective is to use technology to involve patients in spontaneous reporting of ADRs, thus engaging health professionals in regular monitoring of treatment, which improves patient safety and reduces therapy costs. This will boost identification of ADRs at the national level and will open up a new horizon for research to understand and prevent different ADRs. A recently published national study (R.A. Almubark et al., 2020) for community-based adverse drug reactions in Saudi Arabia targeting community pharmacy visitors estimated that over a quarter (28%) of the sample in the KSA experienced symptoms of at least one ADR in the previous year, which is higher than the prevalence in similar studies in Sweden (7.8%) and Italy (9.9%) [26,27]. Consumer reporting could become crucial for the developing countries to implement a proper and efficient PhV system that could reduce morbidity and mortality as well as the economic burden of ADRs [28]. Patients’ participation in the ADR reporting process has been shown to increase spontaneous ADR reporting, which can directly or indirectly minimize hospitalization rates, improve patient health services, increase the safety of medication use, and, as a result, reduce the cost of therapy, advance the national signal detection of ADRs, and encourage the pharmacogenomic studies which will help to understand the risk factors of serious ADRs similar to the SWEDEGENE national study [29,30]. The content analysis of our study revealed that the participants realized the high level of harm that may arise from ADRs and were interested in discussing and obtaining information about them. They expressed their needs to receive an appropriate and prompt care regarding their ADR events. Most participants were not aware of adverse drug reaction reporting systems and they welcomed the use of technology to report ADRs and receive care. While not all the participants had previously had ADRs, they were uncertain about sufficient understanding of ADRs; they mentioned their witnessing of different ADR events with their close family members and friends, in line with the findings of the questionnaire-based study by Ibrahim Sales et al. regarding public awareness of ADRs in Riyadh, KSA [31]. Although the majority of the participants talked about mild-to-moderate ADRs, those who suffered severe ADRs still remembered all details of the events, even the generic or brand name of the possible causal active ingredient. Regarding the occurrence of ADRs, the patients were uncertain whether the new symptoms had been related to their disease or to the newly administered medication. They needed to take a fast action, but they did not know whom they should call to receive the best result.

The most commonly taken action by the majority of them was to stop taking the drug with the highest possibility to cause the event or to stop the treatment which caused the ADRs for sure and could aggravate their health status altogether. Some started to read the pamphlet insert of the medication or scour the Internet; some would rather ask their family physician or pharmacist, look for help at hospitals or pharmacies, or dial the Saudi healthcare phone service “937.” While the participants reported that they proactively asked their healthcare providers for information on their drugs and use them as an ADR resource, most physicians did not actively encourage their patients and consumers to report ADRs. Although the vast majority of health professionals recognize the importance of ADR reporting, their knowledge of the reporting system and reporting of ADRs in Saudi Arabia are insufficient [32]. There is a hotline phone number of the National Drug Information Center of the Saudi Ministry of Health, “937,” which started to deliver pharmaceutical care services and respond to public and professional inquiries in December 2013 [33].

Vander Stichele et al. (1991) concluded that medication package inserts [34] usually provide simple instructions for indications, proper use, dosing, precautions, potential adverse reactions, and comprehensible risk information. These information pieces affect the patients’ understanding, evaluation, and management of medication risks [35]. To date, most patients get information they want about the medications from the drug insert. However, most patients may not read the patient information insert and ultimately become confused about the new side effects they may encounter [15]. The effect of witnessing ADRs on patient understanding of drug usage varies depending on the nature of the incident they have had. Many of them have tried to avoid future use of medication and have opted for taking herbal remedies or other alternative medicines [36]; others agreed that they had to tolerate ADRs as they would have got worse if they quitted the medication [37]. Patients and their caregivers should be educated about the warning signs of potential ADRs, when, who, and how to call and seek help to identify complications. Patient awareness of ADR reporting is vital to improve safety and signal detection. All the patients denied receiving education about ADRs, attending any events about ADRs or rational use of medications; they also denied their awareness of the ADR reporting system in the KSA [38]. Physicians only take the initiative to discuss ADRs after they have happened and that will never benefit patients and may increase the risk of serious adverse effects which, unluckily, may affect the treatment plan and increase the cost and duration of therapy [39].

The only drawback in using this patient online form is the detailed information that the patient should fill which mainly includes medical terms, which makes it difficult to be understood by regular patients with little medical background and so discourages patient self-reporting. Adverse drug reaction reporting is mainly dependent on spontaneous reporting systems that may be paper based (e.g., the UK’s “Yellow Card” system) or based on electronic (online or mobile) applications. Regarding awareness of the ADR reporting system, the results of this study reflect those found in a systematic review of poor patient awareness of ADR reporting systems. It is important to point out that the SFDA system seeks to facilitate the online or telephonic movement of ADR patient reports directly to the system. On the other hand, some systems are mainly targeted at clinicians, which tend not to improve patient awareness about such systems [31,40].

The reference data on the patient needs to receive treatment via ADR reporting technologies from related KSA studies are not available. The majority of the participants supported the view that using technology in ADR reporting would save time and effort, where they would be able to obtain medical services and advice from anywhere and at any moment; however, few objected to the idea of using technology; they did not know how it would help them; they want to meet their healthcare providers face to face. We received participants’ reactions to the concept of having an ADR smartphone application, where they felt relaxed, safe, and efficient, commented that they liked the concept, and welcomed such smartphone application ADR reporting. Some worried about how they would get their necessary care and preferred that the service was supported by or was maintained under the guidance of the Health Ministry. The participants varied in reaction to the cost; the majority of participants would never mind paying either a little or any amount of money to receive a service that would save their lives and save them while using medication, and some would like it to be provided as a free patient care service. There are hundreds of regional and international studies assessing knowledge, attitudes, and perceptions of ADR reporting among health practitioners (physicians, pharmacists, and nurses). Most of these studies demonstrate poor practice, knowledge, and lack of experience in reporting [41]. It was made compulsory for HCPs in Europe by 2012 to report any ADRs suffered by patients, thus improving the practice of ADR reporting. If the quality of ADR reporting by HCPs is poor, how will the patients become aware of it? [42] ADR reporting has dramatically improved at the international level over the last few years, but it is still emerging in the KSA [26,43,44]. Implementation of clinical pharmacy services at hospitals and awareness raising of the role of clinical pharmacists in patient education may contribute to improving ADR reporting and patient safety [45,46]. The key strength of this study is that it was the first population-based assessment of ADR reporting needs and preferences for using technology in the reporting process. Qualitative research has a limitation that data are not mathematically evaluated but rely on opinion and judgment. The minimal analysis includes the risk of collection and recall bias. Further studies should include opinions higher authorities and healthcare providers and institutions towards the possibility of integrating patient-centered ADR reporting technology in the system. The participants were aware of the high level of harm that may arise from adverse drug reactions, which they were eager to discuss and gather information about. They expressed their need to take prompt care of their adverse drug reaction events. Most participants were not aware of adverse drug reaction reporting systems and agreed to the use of technology to report and receive treatment. The widespread use of mobile phones among all genders and ages in the population has opened up space for its integration. The authors assume that educational campaigns to increase awareness of the benefits of ADR reporting would be a step forward in the field of pharmacovigilance. Furthermore, a strict law that requires HCPs to escalate any patient-reported ADRs should be enforced; this can further highlight the role of pharmacists in the field of pharmacovigilance.

## 5. Conclusions

The results of this study show that while the public is eager to gain information about ADRs and take advantage of ADR reporting, their comprehension of ADRs is insufficient and they still need training and support. Thus, the future goal should be to use technology to involve patients in ADR reporting, to increase the national signal identification of ADRs, and to provide a new research pathway for understanding and prevention of various ADRs.

## Figures and Tables

**Table 1:** Interview guide.

I Agree to an Audio-Recorded Interview and the Use Thereof for Research Purposes after My Approval of the Written Interview Transcript. Participant’s Signature: _____________	
**Specific Topic of Investigation**	**Specific Questions**
1. Familiarity with ADRs	1.1. What do you think about adverse drug reactions (ADRs)?
2. Experience with ADRs and actions taken	2.1. Have you or any of your relatives ever experienced ADRs after taking any medication? 2.2. If so, how did you deal with the symptoms? Whom did you contact?
	2.3. If not, could you please tell me the scenario that you think you need to follow to be clear about ADRs?
3. Certainty of the symptoms	3.1. Why did you think that those symptoms were related to your medicine?
	3.2. What evidence did you have to consider the relationship between the unusual symptoms and your medicine?
	3.3. Was it the first time to take this medication?
	3.4. If not, had you been taking this medication for a long time?
	3.5. Were you taking any other medicines regularly at that time?
	3.6. What were the other causes that you suspected?
	3.7. Did you confirm the suspected symptoms with any information sources?
4. Previous education regarding ADRs	4.1. Did you receive any education regarding the expected ADRs you may experience from certain medication or advice regarding the ways to alleviate any effects, how to deal with them, or who to contact? 4.2. Did you attend any public awareness events or webinars related to ADRs?
5. Awareness of the ADR reporting system	5.1. Do you feel that patients may need a certain authority to which they can report their ADRs? 5.2. Do you know that you can report your ADRs? 5.3. Do you know the benefit of reporting your ADRs? Are you aware to whom you can report your ADRs?
	5.4. What do you know about the SFDA reporting system?
6. Role of information technology in ADR reporting	6.1. What do you think about the role of information technology (websites, social media, smartphone applications…) in patient communication with the health authorities and in improving health services? 6.2. Do you think that information technology can help patients in ADR reporting?
	If not, why?
7. Use of mobile applications for ADR reporting	7.1. What do you think about the use of smartphone applications for ADR reporting? Friendly, understandable, or simple. 7.2. What do you prefer to have, a free or paid ADR reporting service?

**Table 2:** Example of coding process.

Meaning Unit	Condensed Meaning Unit	Code	Subtheme	Theme
“Mostly side effects like dryness or dizziness and some of them are vomiting or sometimes incorrect use of the drug.” “ADRs mean unwanted noxious effects.”	Normal side effect	Adverse drug reactions	ADR definition	Familiarity with ADRs

**Table 3:** Demographic data of the participants.

Characteristics	*N* = 15	%
**Gender**		
Male	6	40
Female	9	60
**Age**		
18–24	2	13
25–35	6	40
36–45	3	20
46–55	1	7
56–65	3	20
**Marital status**		
Single	7	46.67
Married	8	53.33
**Educational level**		
Primary school	1	6.7
Secondary school	3	2
College or more	11	73.3
**Occupational status**		
Full-time	6	40
Part-time	2	13.33
Unemployed	7	46.67
**Monthly income**		
Less than 5000 SR *	6	40
5000–10,000 SR	6	40
More than 10,000 SR	3	20

* SR (1 Saudi riyal equals 0.27 United States dollars).

## Data Availability

Data supporting the findings of this study are available from the respective author on request. The information is not available to the public due to privacy or legal constraints.

## Abbreviations

ADRs	Adverse drug reactions
WHO	World Health Organization
MHRA	Medicines and Healthcare products Regulatory Agency
UMC	Uppsala Monitoring Center
SPAs	Smartphone applications
FDA	Food and Drug Administration
SFDA	Saudi Food and Drug Authority
SRS	Spontaneous reporting systems
EU	European Union
PhV	Pharmacovigilance
KSA	Kingdom of Saudi Arabia
HCP	Healthcare professional
IT	Information technology
UK	United Kingdom
